# Methylation of *ESR1* promoter induced by SNAI2–DNMT3B complex promotes epithelial–mesenchymal transition and correlates with poor prognosis in ERα‐positive breast cancers

**DOI:** 10.1002/mco2.403

**Published:** 2023-10-24

**Authors:** Ji‐Wei Li, Qiu‐Min Deng, Jian‐Ling Zhu, Wei Min, Xiao‐Yi Hu, Hong‐ Yu Chen, Zhong Luo, Lin‐Ling Lin, Xiao‐Long Wei, Yong‐Qu Zhang, Kang‐Liang Lou, Yi‐Yang Gao, Guo‐Jun Zhang, Jing‐Wen Bai

**Affiliations:** ^1^ Fujian Key Laboratory of Precision Diagnosis and Treatment in Breast Cancer Xiang'an Hospital of Xiamen University School of Medicine Xiamen University Xiamen P. R. China; ^2^ Department of Respiratory, Critical Care and Sleep Medicine Xiang'an Hospital of Xiamen University, School of Medicine, Xiamen University Xiamen P. R. China; ^3^ Xiamen Key Laboratory of Endocrine‐Related Cancer Precision Medicine Xiang'an Hospital of Xiamen University School of Medicine Xiamen University Xiamen P. R. China; ^4^ Xiamen Research Center of Clinical Medicine in Breast and Thyroid Cancers Xiang'an Hospital of Xiamen University School of Medicine Xiamen University Xiamen P. R. China; ^5^ Department of Breast‐Thyroid‐Surgery and Cancer Center Xiang'an Hospital of Xiamen University School of Medicine Xiamen University Xiamen P. R. China; ^6^ Department of Pathology Cancer Hospital of Shantou University Medical College Shantou P. R. China; ^7^ Cancer Research Center of Xiamen University School of Medicine Xiamen University Xiamen P. R. China; ^8^ Department of Oncology Xiang'an Hospital of Xiamen University School of Medicine Xiamen University Xiamen P. R. China

**Keywords:** breast cancer, DNA methyltransferase 3 beta, epithelial–mesenchymal transition, ESR1 methylation, snail family transcriptional repressor 2

## Abstract

Estrogen receptor α (ERα) serves as an essential therapeutic predictor for breast cancer (BC) patients and is regulated by epigenetic modification. Abnormal methylation of cytosine phosphoric acid guanine islands in the estrogen receptor 1 (ESR1) gene promoter could silence or decrease ERα expression. In ERα‐negative BC, we previously found snail family transcriptional repressor 2 (SNAI2), a zinc‐finger transcriptional factor, recruited lysine‐specific demethylase 1 to the promoter to transcriptionally suppress ERα expression by demethylating histone H3 lysine 4 dimethylation (H3K4me2). However, the role of SNAI2 in ERα‐positive BC remains elusive. In this study, we observed a positive correlation between SNAI2 and *ESR1* methylation, and SNAI2 promoted *ESR1* methylation by recruiting DNA methyltransferase 3 beta (DNMT3B) rather than DNA methyltransferase 1 (DNMT1) in ERα‐positive BC cells. Subsequent enrichment analysis illustrated that *ESR1* methylation is strongly correlated with cell adhesion and junction. Knocking down DNMT3B could partially reverse SNAI2 overexpression‐induced cell proliferation, migration, and invasion. Moreover, high DNMT3B expression predicted poor relapse‐free survival and overall survival in ERα‐positive BC patients. In conclusion, this study demonstrated the novel mechanisms of the *ESR1* methylation mediated with the SNAI2/DNMT3B complex and enhanced awareness of *ESR1* methylation's role in promoting epithelial–mesenchymal transition in BC.

## Graphical abstract

In an ERα‐positive BC cell, SNAI2 recruited DNMT3B to form a SNAI2‐DNMT3B combination and bound on the E‐box region of the *ESR1* promoter to stimulate promoter methylation. The elevated *ESR1* methylation resulted in a condensed chromatin state, transcriptional repression, and gene silencing of *ESR1*. Besides, SNAI2‐DNMT3B‐*ESR1* also significantly facilitated the process of epithelial–mesenchymal transition and promoted the invasiveness of ERα‐positive BC cells.

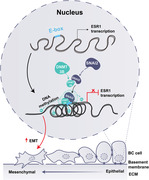

## 1 INTRODUCTION

Breast cancer (BC) is women's most commonly diagnosed malignancy worldwide.^1^ As a heterogeneous disease,^2^ it contains distinct molecular and genetic subtypes with different molecular alterations, histologic behavior, and clinical outcomes. Around two‐thirds of BC patients express estrogen receptor α (ERα), associated with well‐differentiated tumors, a good response to endocrine therapy, and a favorable prognosis.^3^ However, a large meta‐analysis reported that 20% of the ERα‐positive BC lost ERα expression during disease progression and developed resistance to hormone treatment and further distant metastases.^4^

The molecular mechanisms underlying ERα deficiency include transcriptional repression, epigenetic regulation, post‐transcriptional regulation, and genetic mutation.^5^ Epigenetic regulation mainly includes DNA methylation, histone modifications, nucleosome remodeling, and regulation by noncoding RNAs.^6^ DNA methylation was a process of adding a methyl group to the fifth carbon position of cytosine residue in a cytosine phosphoric acid guanine (CpG) island, a cytosine‐ and guanine‐rich area in the promoter. Abnormal methylation of the CpG islands located in promoter regions of estrogen receptor 1 (*ESR1*)

was responsible for 41% of cases of ERα absence.^7^ CpG islands were hypomethylated in ERα‐positive cell lines like MCF‐7, ZR75‐1, and T47D, whereas they were hypermethylated in ERα‐negative BC cells such as MDA‐MB‐231, MDA‐MB‐435, MDA‐MB‐468, and Hs578t.^8^

Promoter methylation is catalyzed by DNA methyltransferases (DNMTs), which include DNMT1, DNMT3A, and DNMT3B.^9^ DNMT1 maintained methylation of hemimethylated DNA, while DNMT3A/3B was mainly involved in *de novo* methylation. Namely, DNMT3A/3B had high activity on unmethylated substrates.^10^, ^11^ DNMT3B was frequently implicated in normal physiological development and cancer genetic mutations. Recent emerging evidence indicated that epigenetic alteration caused by DNMT3B abnormalities, like TCF3 silencing in endometrial cancer^12^ and CTH downregulation in hepatocellular carcinoma,^13^ was associated with tumorigenesis and tumor progression. Demethylation could reactivate *ESR1* expression in ERα‐negative BC cells.^14^ It was reported that treatment with the DNMT inhibitor 5‐aza‐2′‐deoxycytidine (5‐AZA) restored ERα expression and attenuated cell growth in MDA‐MB‐231 and Hs578t cells.^15^ It had been shown that DNMT1 and DNMT3B were associated with hypermethylation of the *ESR1* promoter in MDA‐MB‐231 cells, whereas DNMT3A showed little association.^15^ Inhibition of DNMT1^16^ or DNMT3B^17^ by antisense oligonucleotides caused *ESR1* gene re‐expression and transcriptional activation in MDA‐MB‐231 or MCF‐7 with ZEB1 overexpression.

Meanwhile, the correlations between ERα expression loss and epithelial–mesenchymal transition (EMT), invasion, and metastasis have been explored in multiple types of research.^18^, ^19^, ^20^ Snail family transcriptional repressor 2 (SNAI2), also called SNAIL2 or Slug, is a classical EMT‐related transcriptional inhibitor with an *N*‐terminal SNAG domain and a C‐terminal DNA‐binding domain,^21^, ^22^, ^23^, ^24^ was negatively associated with ERα expression in both BC and lung cancer.^25^, ^26^ As previously reported, SNAI2 could suppress *ESR1* gene expression by forming a complex with lysine‐specific demethylase 1 (LSD1). Mechanistically, SNAI2 bound to the E‐boxes in the *ESR1* promoter and further recruited epigenetic modifier LSD1 to inhibit *ESR1* transcription only in the human triple‐negative BC.^27^ Another study proved that SNAI2 downregulated ERα expression indirectly by targeting miR‐221.^28^ On the other hand, ERα appeared to inhibit SNAI2 expression by directly repressing SNAI2 transcription^29^ or indirectly mediating SNAI2 degradation.^25^ More specifically, it had been discovered that a complex of HDAC1, nuclear receptor co‐repressor, and activated ERα could bind to the *SNAI2* promoter and decrease transcription of *SNAI2*.^29^ Moreover, the PI3K/Akt/GSK‐3β pathway was activated by ERα and led to GSK‐3β phosphorylation and subsequent proteasomal degradation of SNAI2 via ubiquitination.^25^

In this study, we explored the role of *ESR1* methylation induced by the SNAI2‐DNMT3B complex and its association with the clinical outcome or clinicopathological characteristics in BC, especially in ERα‐positive BC. We also investigated whether this mechanism of regulation correlated with EMT in BC. Our study displays progress in understanding the contribution of *ESR1* methylation in EMT and BC prognosis and its capability to provide a potential target for epigenetic‐based BC therapy.

## 2 RESULTS

### 2.1 SNAI2 promoted ESR1 promoter methylation in ERα‐positive BC

**FIGURE 1:**
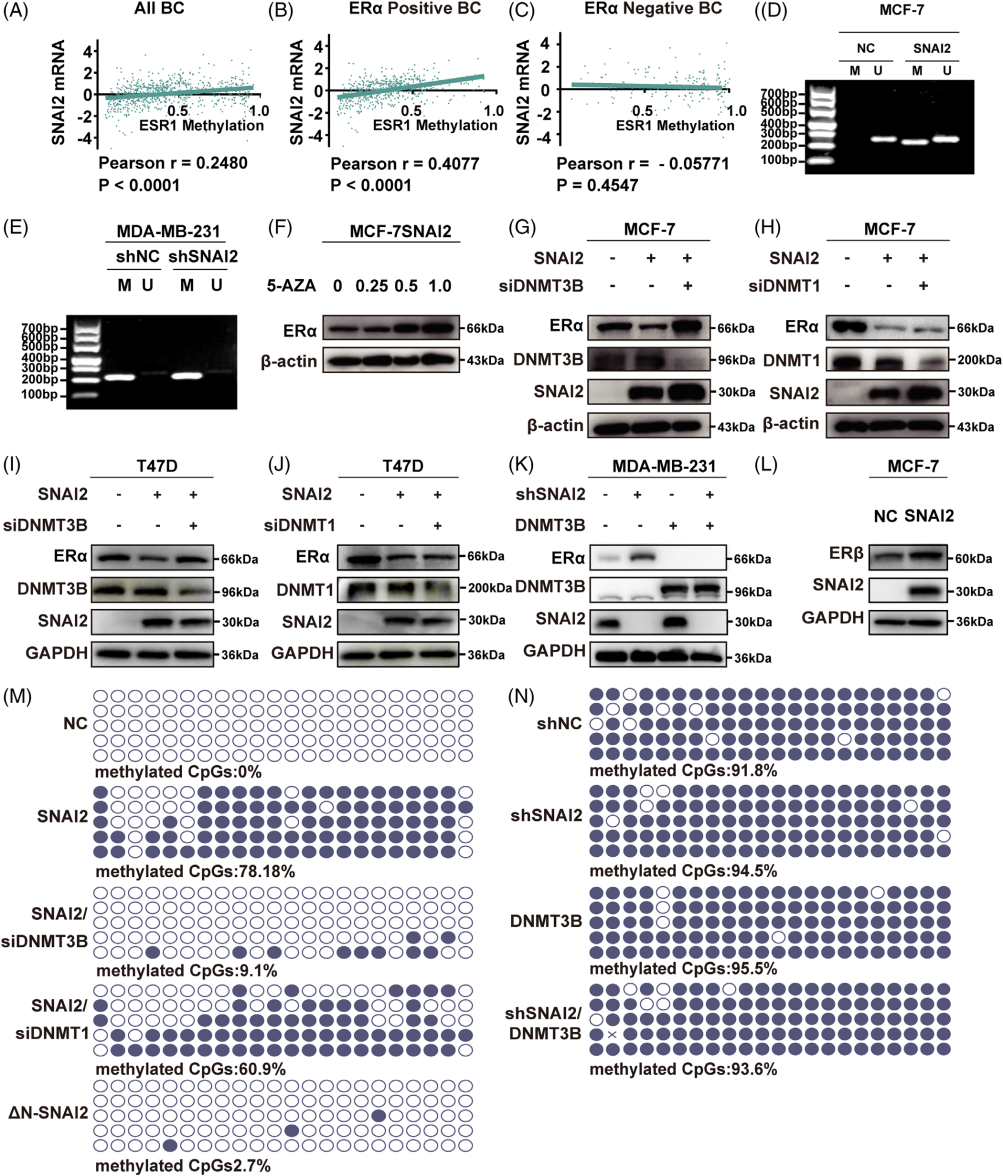
Positive correlation between SNAI2 and *ESR1* methylation in human BC. (A‐C) Correlation analysis of SNAI2 and *ESR1* methylation based on z‐scores of SNAI2 mRNA levels for all BC (n = 1108, A), ER‐positive cases (n = 573, B), and ER‐negative cases (n = 170, C) by two‐tailed Pearson's *R* tests. Z‐scores of SNAI2 mRNA were directly obtained from online data (www.cbioportal.org). The formula is z = (expression in tumor sample—mean expression in reference sample)/standard deviation of expression in the reference sample. (D) MSP analysis to detect the methylation status of *ESR1* promoter in MCF‐7SNAI2 and its control group. M, Methylated; U, Unmethylated. (E) MSP analysis to see the methylation status of *ESR1* promoter in MDA‐MB‐231shSNAI2 and its control group. M, Methylated; U, Unmethylated. (F) Western blot analysis of ERα expression levels in MCF‐7SNAI2 cells treated with varying concentrations of DNA methylation inhibitor 5‐AZA (0, 0.25, 0.5, and 1 M). (G) Western blot analysis of ERα expression in SNAI2‐overexpressed MCF‐7 cells with or without DNMT3B knockdown. (H) Western blot analysis of ERα expression in SNAI2‐overexpressed MCF‐7 cells with or without DNMT1 knockdown. (I) Western blot analysis of ERα expression in SNAI2‐overexpressed T47D cells with or without DNMT3B knockdown. (J) Western blot analysis of ERα expression in SNAI2‐overexpressed T47D cells with or without DNMT1 knockdown. (K) Western blot analysis of ERα expression in MDA‐MB‐231 cells to verify the effects of SNAI2 and DNMT3B via SNAI2 knockdown and DNMT3B overexpression. (L) Western blot analysis of ERβ expression in SNAI2‐overexpressed MCF‐7 cell and its control group. (M) BSP analysis to detect hypermethylated CpG sites in MCF‐7NC cells, MCF‐7SNAI2 cells, MCF‐7SNAI2/siDNMT3B cells, MCF‐7SNAI2/siDNMT1 and MCF‐7 ΔN‐SNAI2 cells. (N) BSP analysis to detect hypermethylated CpG sites in MDA‐MB‐231shNC cells, MDA‐MB‐231shSNAI2 cells, MDA‐MB‐231DNMT3B cells, and MDA‐MB‐231shSNAI2/DNMT3B cells. Transfection for Western blot analysis was performed in six‐well dishes. The indicated plasmid or siRNA amounts were 3 μg for plasmid and 50 nM for siRNA per well. MSP and BSP analyses were transfected in 60‐mm dishes, and the amounts of plasmid and siRNA were 6 μg and 100 nM per well, respectively.

As we reported previously, SNAI2 transcriptionally repressed *ESR1* gene expression in BC. Abnormal DNA methylation can lead to gene silencing at the transcriptional level. We utilized TCGA data to examine the correlation between SNAI2 expression and *ESR1* methylation. We observed a statistically significant positive correlation between SNAI2 and *ESR1* methylation in BC patients (Pearson *r* = 0.2480, *p* < 0.0001), especially in ERα‐positive (Pearson *r* = 0.4077, *p* < 0.0001) rather than ER‐negative BC patients (Pearson *r* = −0.05771, *p* = 0.4547) (Figure 1A‐C).

To explore the relationship between *ESR1* methylation and SNAI2 in BC cell lines, we also searched the CpG island prediction database, MethPrime. Then, we found a 256‐bp CpG‐rich region (−703 to −448 bp) in the *ESR1* promoter (−910 to +90 bp). The methylation status of the *ESR1* promoter was detected using methylation‐specific PCR (MSP) analysis in SNAI2‐overexpressed stable BC cell lines MCF‐7SNAI2 and their control cells. As seen in Figure 1D, a methylated band appeared in SNAI2‐overexpressed MCF‐7 cells. Unexpectedly, no significant change was found in the stable SNAI2 knocking down MDA‐MB‐231shSNAI2 and its control cells (shNC) (Figure 1E). Then, we treated MCF‐7SNAI2 cells with 5‐AZA in concentrations of 0, 0.25, 0.5, and 1 μM. The results indicated that 5‐AZA reversed SNAI2‐mediated downregulation of ERα (Figure 1F) in a dose‐dependent manner. In brief, SNAI2 represses ERα expression by stimulating *ESR1* promoter methylation in ERα‐positive BC.

To explore the role of DNA methyl transferase (DNMT1 or DNMT3B) in SNAI2‐induced upregulation of *ESR1* methylation, the DNMT1 or DNMT3B‐specific short hairpin RNA (siRNA) was introduced into MCF‐7SNAI2 cells, respectively. We discovered that ERα was rescued in a DNMT3B‐dependent (Figure 1G) and DNMT1‐independent (Figure 1H) manner at the protein level. A similar tendency was observed in T47D cells as well (Figure 1I, J). From Figure 1K, in the MDA‐MB‐231 cell line, we found that overexpression of DNMT3B led to the downregulation of ERα. However, the simultaneous overexpression of DNMT3B and interference with SNAI2 still downregulate ERα compared with the control group, suggesting that DNMT3B could not collaborate with SNAI2. In addition, we also investigate the expression of ERβ, which is the isoform of ERα but often functions differently from ERα in modulating significant phenotypic changes.^30^, ^31^ In this study, ERβ was upregulated after SNAI2 overexpression in MCF‐7 cells (Figure 1L).

We employed a quantitative methylation assessment technology, Bisulfite sequencing PCR (BSP), to directly pinpoint the effect of DNMT1 or DNMT3B on the ESR1 methylation level. None of the methylated CpG sites were detected in MCF‐7NC cells, whereas around 78.18% hypermethylation was observed in the MCF‐7SNAI2 cell line. When DNMT3B was knocked down, only 9.1% hypermethylation was observed. By contrast, there is a slight change in DNA methylation status after DNMT1 knockdown in MCF‐7SNAI2 cells (Figure 1M). In the meantime, we performed BSP in MDA‐MB‐231 cells and found hypermethylation status was maintained even in the shSNAI2 group compared with the control group (methylated CpGs: 94.5 vs. 91.8%, Figure 1N). And DNMT3B overexpression in MDA‐MB‐231shSNAI2 cells (shSNAI2/DNMT3B) did not distinctly impact the hypermethylation status of the *ESR1* promoter (methylated CpGs: 93.6 vs. 94.5%, Figure 1N). In MSP analysis, no significant difference was found between the band of the shSNAI2/DNMT3B and the shSNAI2 group, either (Supporting Information Figure S1). Collectively, these data implied that SNAI2 promoted *ESR1* methylation in ERα‐positive BC cells through DNMT3B.

### 2.2 SNAI2 recruited DNMT3B to repress ESR1 transcription

**FIGURE 2:**
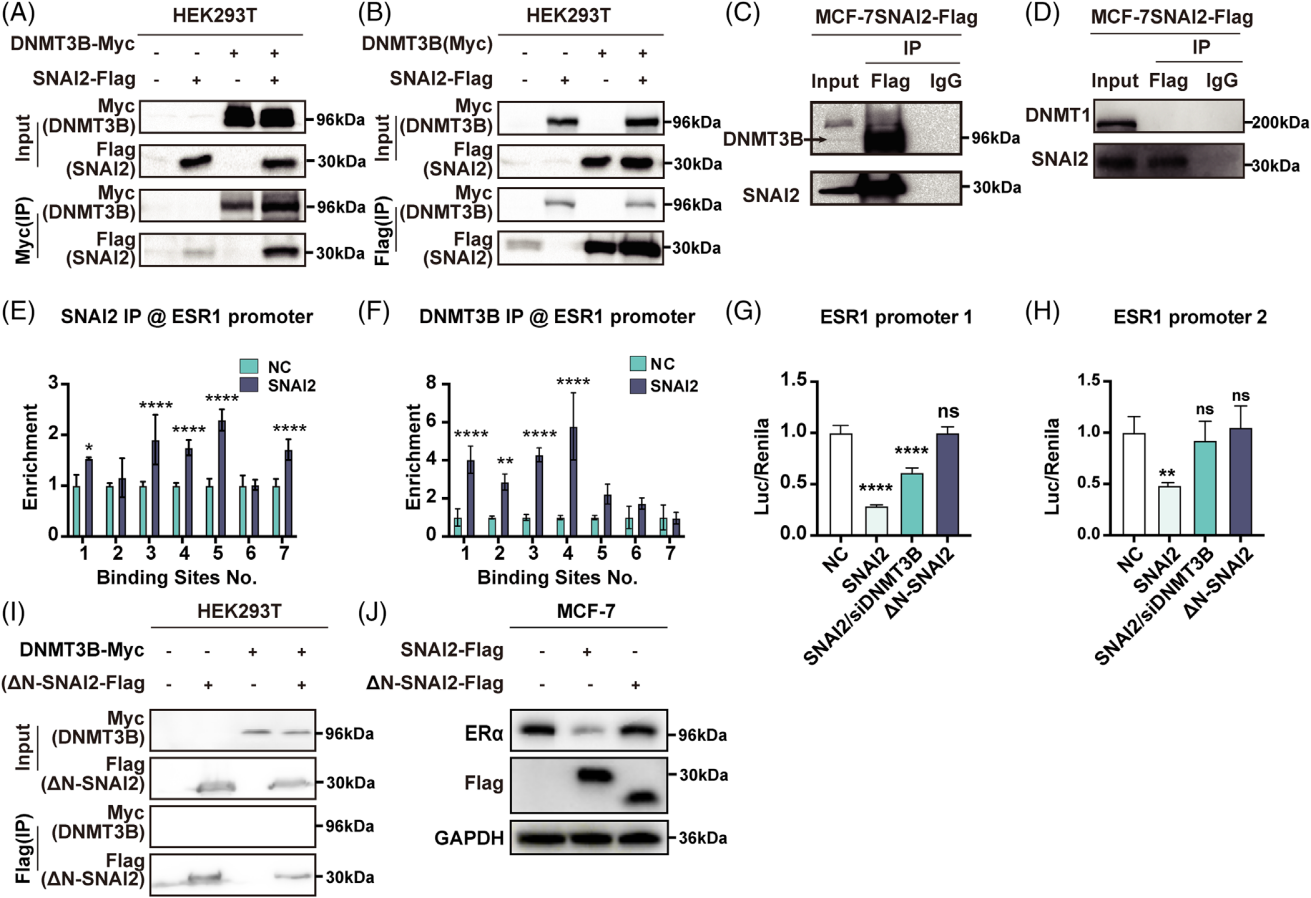
SNAI2 recruited DNMT3B to repress ESR1 expression transcriptionally. (A‐B) Co‐IP analysis using anti‐Myc (A) or anti‐Flag antibody (B) in HEK293T cells expressing SNAI2‐Flag and DNMT3B‐Myc or SNAI2‐Flag /DNMT3B‐Myc. (C) IP analysis using an antibody specific to Flag in MCF‐7 cell line that overexpressed FLAG‐tagged SNAI2. Expression of DNMT3B in the immunocomplexes was detected by DNMT3B‐specific antibody. (D) IP analysis using an antibody specific to flag in MCF‐7 cell line that overexpressed FLAG‐tagged SNAI2. Expression of DNMT1 in the immunocomplexes was detected by DNMT1‐specific antibody. (E) ChIP analysis to determine the E‐box binding sites SNAI2 occupied in the *ESR1* promoter in MCF‐7 cells. Seven primer pairs were designed to cover different binding sites within *ESR1* promoters 1 and 2. (F) ChIP analysis to determine E‐box binding sites that DNMT3B occupied in the *ESR1* promoter in MCF‐7SNAI2 cells. (G‐H) Dual luciferase reporter assay to compare the transcriptional activity of *ESR1* promoter 1 (I) and *ESR1* promoter 2 (J) in MCF‐7 cells, SNAI2‐overexpressed MCF‐7 cells with or without DNMT3B knockdown, and ΔN‐SNAI2 overexpressed MCF‐7 cells. (I) co‐IP analysis using anti‐Flag antibodies in HEK293T cells expressing ΔN‐SNAI2‐Flag, DNMT3B‐Myc or ΔN‐SNAI2‐Flag/DNMT3B‐Myc. (J) Western blot analysis of ERα expression in SNAI2 or ΔN‐SNAI2 overexpressed MCF‐7 cells. Data are presented as the mean ± SD of at least three independent experiments. *p < 0.05, **p < 0.01, ***p < 0.001, and ****p < 0.0001 (Student's *t*‐test) as compared to control cells. Transfections for co‐IP and IP analysis were performed in 100‐mm dishes, and the corresponding plasmids were 15 μg each.

To confirm the interaction between SNAI2 and DNMT3B, a co‐immunoprecipitation (Co‐IP) assay was conducted in the HEK293T cell line transiently co‐transfected with Flag‐tagged SNAI2 and Myc‐tagged DNMT3B. The SNAI2 formed a complex with DNMT3B (Figure 2A,B). We repeated the IP assay in the MCF‐7SNAI2 cell line that overexpressed FLAG‐tagged SNAI2 and found endogenous DNMT3B was immunoprecipitated by Flag (Figure 2C), whereas DNMT1 was not efficiently precipitated in the same experimental condition (Figure 2D).

SNAI2 transcriptionally repressed gene expression by binding to E‐box (5′‐CANNTG‐3′) motifs in the promotor region. There are eight potential SNAI2‐binding sites in the *ESR1* promoter (2 500‐bp upstream and 500‐bp downstream of the transcription start site). *ESR1* promoter was divided into three parts: *ESR1* promoter 1 covered the first three, *ESR1* promoter 2 covered the fourth through seventh, and *ESR1* promoter 3 covered the last binding site in our previous report. We have proven that SNAI2 overexpression significantly reduced *ESR1* promoter 1 and 2 activity while having a negligible impact on the *ESR1* promoter 3 in MCF‐7 cells in our previous report. Therefore, the first seven binding sites were analyzed in the following experiment.

To determine whether SNAI2 could bind to the *ESR1* promoter in an E‐box‐dependent manner, the chromatin immunoprecipitation (ChIP)‐qPCR assays were performed in SNAI2‐overexpressed and controlled MCF‐7 cells. The amounts of SNAI2 binding to E‐boxes of the *ESR1* promoters increased when compared with control cells in the first, third, fourth, fifth, and seventh binding sites (Figure 2E). We next tried to address the ability of DNMT3B to bind E‐boxes in MCF‐7 cells using an anti‐DNMT3B antibody. Significant enrichment of DNMT3B was observed at four (first, second, third, and fourth) putative binding sites in the *ESR1* promoter 2 regions when SNAI2 was overexpressed (Figure 2F). These data implied that SNAI2 binds directly or as part of a SNAI2‐DNMT3B complex to the *ESR1* promoters 1 and 2.

Moreover, to delineate the transcriptional activity of the SNAI2‐DNMT3B complex to the *ESR1* promoter, we conducted a dual luciferase reporter assay in the MCF‐7 cell line. As shown in Figure 2G,H, the activity of *ESR1* promoters 1 and 2 was significantly repressed by overexpressing SNAI2, and knocking down DNMT3B reversed this repression.

Our previous results showed that the mutant ΔN‐SNAI2 lacking the SNAG domain could not interact with the chromatin‐modifying protein LSD1. We wanted to explore if the SNAG domain was essential for SNAI2 to combine with DNMT3B. In a Co‐IP assay conducted in the HEK293T cell line, Flag‐tagged ΔN‐SNAI2 could not pull down the Myc‐tagged DNMT3B (Figure 2I).

To investigate the effect of the SNAG domain for repressing *ESR1* expression, we found no significant change in methylated CpGs sites in the *ESR1* promoter in the ΔN‐SNAI2 group compared with the control group cells (2.7 vs. 0%) (Figure 1M). In the dual luciferase reporter assay, we found that enforced expression of ΔN‐SNAI2 did not affect the activity of *ESR1* promoters 1 and 2 (Figures 2G,H). Overexpression of a mutant ΔN‐SNAI2 did not impact ERα expression (Figure 2J). Taken together, the SNAG domain was essential for combining SNAI2 and DNMT3B and regulating *ESR1* methylation and ERα expression.

### 2.3 ESR1 methylation had a strong relationship with EMT

**FIGURE 3:**
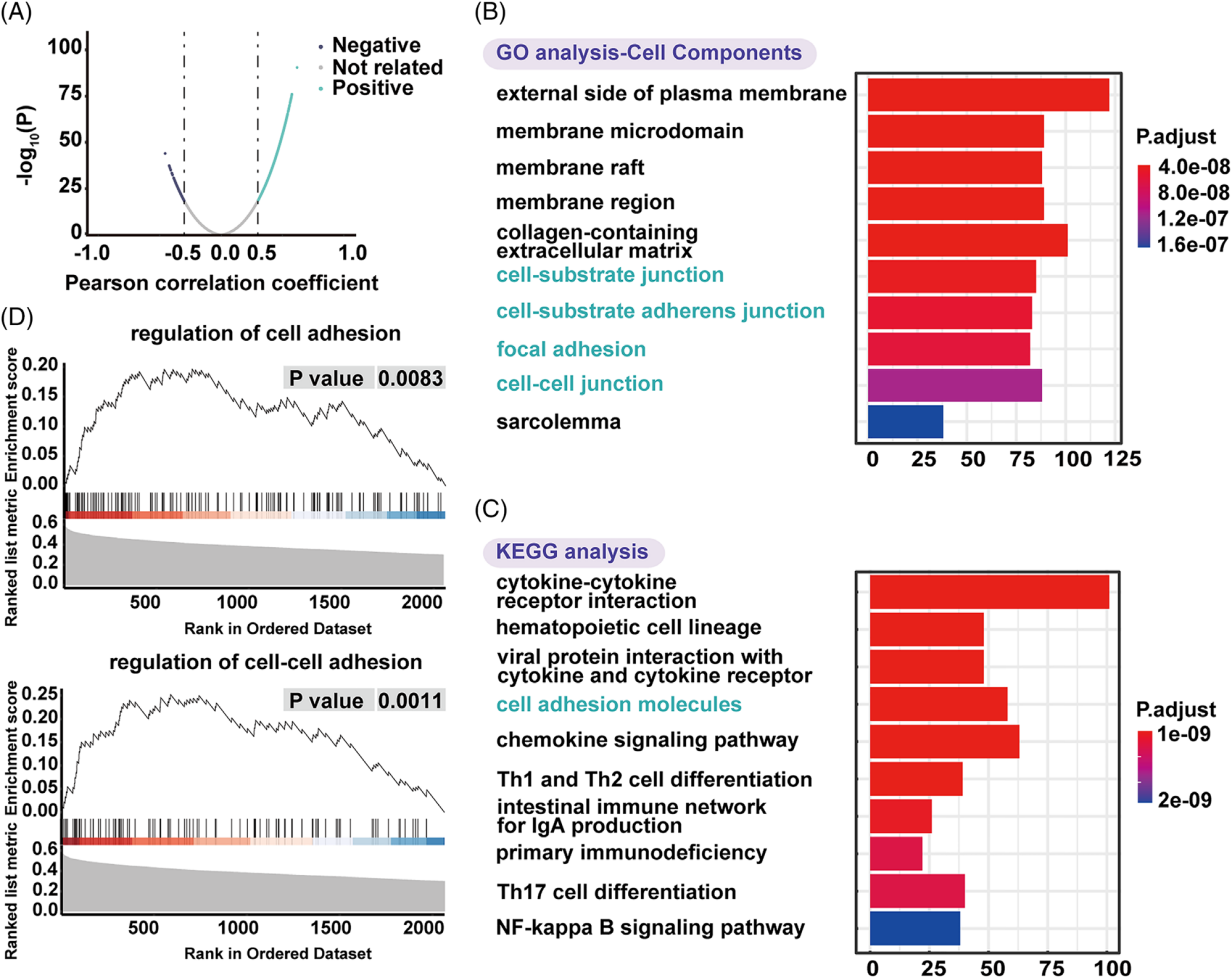
Relationship between *ESR1* methylation with EMT (A) Volcano plot distribution of the RNA‐array data. (B) GO analysis of cell components for *ESR1* methylation‐related genes whose mRNAs retain a |Pearson correlation coefficient| ≥ 0.3 (C) KEGG pathway analysis for *ESR1* methylation‐related genes. (D) GSEA analysis of cell adhesion (upper)‐ and cell‐cell adhesion (below)‐related genes for *ESR1* methylation‐related genes.

To further explore the biological function of *ESR1* methylation, gene ontology (GO) function, and Kyoto encyclopedia of genes and genomes (KEGG) pathway analysis were conducted. mRNAs with a |Pearson correlation coefficient| ≥ 0.3 were considered *ESR1* methylation‐related genes. A total of 2483 *ESR1* methylation‐related mRNAs were visualized by a volcanic map (Figure 3A). They were significantly enriched in biological processes, including T‐ and lymphocyte activation, cell‐cell adhesion, lymphocyte differentiation (Supporting Information Figure S2); cellular components (Figure 3B), such as cell‐cell junction, cell‐substrate junction, cell‐substrate adherens junction, and focal adhesion, which was deeply related with EMT; molecular functions (Supporting Information Figure S2), such as receptor‐ligand activity, glycosaminoglycan binding, cytokine receptor binding, *etc*. The KEGG analysis showed enrichment of *ESR1* methylation‐related mRNAs in cell adhesion molecules (Figure 3C). In gene set enrichment analysis (GSEA), *ESR1* methylation‐related genes were highly enriched in regulating cell adhesion and cell‐cell adhesion (Figure 3D). Collectively, *ESR1* methylation showed a strong relationship with EMT.

### 2.4 Knockdown of DNMT3B impaired SNAI2's role in promoting EMT and proliferation

**FIGURE 4:**
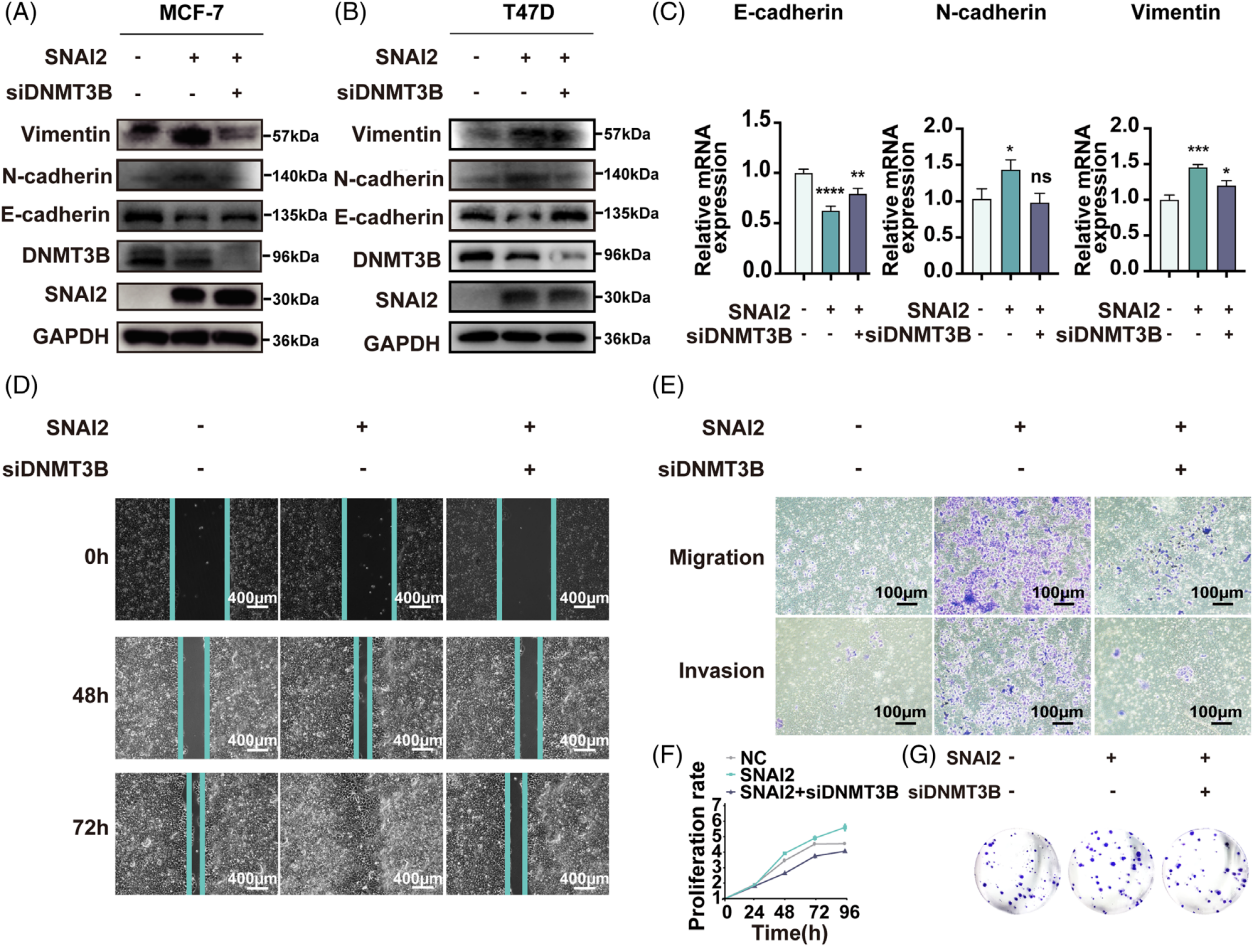
Knockdown of DNMT3B impaired SNAI2's role in promoting migration, invasion, and cell proliferation ability. (A‐B) Western blot analysis of E‐cadherin, N‐cadherin, and Vimentin expression in SNAI2‐overexpressed MCF‐7 (A) and T47D (B) cells with or without DNMT3B knockdown. (C) RT‐PCR analysis of E‐cadherin, N‐cadherin, and Vimentin expression in SNAI2‐overexpressed MCF‐7 cells with or without DNMT3B knockdown. (D) Wound healing assay of MCF‐7SNAI2 and MCF‐7SNAI2 cells with DNMT3B knockdown. Representative images are shown. Magnification, 100X. (E) Migration and invasion assays in MCF‐7SNAI2 and MCF‐7SNAI2 cells with DNMT3B knockdown. Representative images of migrated (upper) and invaded (lower) cells were shown. Magnification, 400×. (F) Comparison of proliferative ability in MCF‐7SNAI2 and MCF‐7SNAI2 cells with DNMT3B knockdown. (G) Colony formation assay of MCF‐7SNAI2 cells and MCF‐7SNAI2 cells with DNMT3B knockdown. Transfections were all performed in six‐well dishes. The indicated plasmid or siRNA amounts were 3 μg for SNAI2‐overexpressing plasmid and 50 nM for DNMT3B siRNA per well. Data are presented as mean ± SD of at least three independent experiments. *p < 0.05, **p < 0.01, ***p < 0.001, and ****p < 0.0001 (Student's *t*‐test) as compared to control cells.

Bioinformatics analysis based on GO function, KEGG pathway, and GSEA analysis showed that *ESR1* methylation significantly correlated with EMT. In Western blot analysis, EMT‐related markers, like E‐cadherin, N‐cadherin, and Vimentin, were rescued in a DNMT3B‐dependent manner both in MCF‐7 (Figure 4A) and T47D (Figure 4B) cell lines. qRT‐PCR analysis of MCF‐7 (Figure 4C) also revealed similar results in SNAI2‐induced EMT.

Wound healing, migration and invasion, cell proliferation, and colony formation assays were also conducted in MCF‐7SNAI2, MCF‐7SNAI2/siDNMT3B cell lines, and their corresponding control groups to verify these functional changes. The wound healing assay measures the migration ability of the MCF‐7 cell line (Figure 4D). The percent of wound closure relative to the initial scratch width was about 57.9% in the control group and 78.9% in the MCF‐7SNAI2 group. The ratio reduced to 58.0% after knocking down DNMT3B in SNAI2‐overexpressed cells, indicating that DNMT3B can mediate increased cell motility induced by SNAI2. At 72 h, we obtained similar results that DNMT3B silencing partially rescued ectopic SNAI2‐induced enhancement of cell migration ability. Quantitative analysis is shown in Supporting Information Figure S3A,B.

In the transwell assays, the number of MCF‐7SNAI2 that migrated through the transwell pores was 5.7 times greater than that of control cells after culture for 48 h. While the ratio of migrated cells in the SNAI2/siDNMT3B group decreased by 82.4% compared with the MCF‐7SNAI2 cells, indicating that the upregulation of migration caused by SNAI2 was abolished by accessional knockdown of DNMT3B (Figure 4E upper, Supporting Information Figure S3C). Similar tendencies were shown in invasion assays after culture for 72 h (Figure 4E lower, Supporting Information Figure S3D).

In the cell proliferation assay, MCF‐7 cells transfected with the SNAI2 plasmid grew faster than cells in the control group, and their proliferation capacity was impaired by the knockdown of DNMT3B (Figure 4F). In the colony formation assay, the number of colonies increased by 90.6% in the SNAI2‐overexpression group. It was rescued to 90.1% in the group of DNMT3B knockdown with SNAI2 overexpression compared to the control group (Figure 4G, Supporting Information Figure S3E). These results indicated that SNAI2 and DNMT3B were highly coordinated to regulate BC cells' migration, invasion, and proliferative ability, possibly caused by *ESR1* methylation.

### 2.5 DNMT3B correlated with some clinicpathological factors and predicted a poor prognosis in ERα‐positive BC patients

To investigate the relationship between DNMT3B expression and clinic‐pathological data, we analyzed 1025 BC samples from TCGA. We found that in ERα‐positive BC patients, high expressed DNMT3B means a higher proportion of T2—T4 tumor size (62.3 vs. 52.3%, *p* < 0.01), N1—N3 lymph node status (37.7 vs. 34.2%, *p* < 0.01), II‐IV AJCC stage (57.5 vs. 52.9%, *p* < 0.01), HER2‐positive patients (25.0 vs. 12.4%, *p* < 0.01), and lower proportion of PR‐positive patients (79.5 vs. 88.0%, *p* < 0.01) than the DNMT3B low expression group (Table 1). While in ERα‐negative BC patients, only a higher proportion of I‐I‐IV AJCC stage (64.1 vs. 53.5%, *p* < 0.05), a lower proportion of PR‐positive patients (3.6 vs. 20.9%, *p* < 0.01), and metastasis (2.1 vs. 9.3%, *p* < 0.01) were found to correlate with high expressed DNMT3B (Table 1).

**TABLE 1:** Relationship between DNMT3B expression and clinicopathological parameters in ERα‐positive and ‐negative BC patients, respectively.

	ERα‐Positive				ERα‐Negative			
	DNMT3B Expression (%)				DNMT3B Expression (%)			
Features	Low expression (n = 482)	High expression (n = 332)	χ^2^	*p*	Low expression (n = 43)	High expression (n = 195)	χ^2^	*P*
**Age**								
≤50	129 (26.8%)	100 (30.1%)	1.06	0.30	13 (30.2%)	74 (37.9%)	0.90	0.34
>50	352 (73.0%)	232 (69.9%)			30 (69.8%)	121 (62.1%)		
N/A	1 (0.2%)	0 (0.0%)						
**Tumor size**								
T1	144 (29.9%)	71 (21.4%)	7.26	0.007	13 (30.2%)	41 (21.0%)	1.66	0.20
T2–T4	337 (52.3%)	260 (62.3%)			30 (53.5%)	153 (65.1%)		
N/A	1 (0.2%)	1 (0.3%)			0 (0.0%)	1 (0.5%)		
**Nodal status**								
N0	231 (47.9%)	125 (37.7%)	8.03	0.0046	27 (62.8%)	109 (55.9%)	1.38	0.24
N1–N3	243 (34.2%)	199 (37.7%)			14 (25.6%)	86 (26.7%)		
N/A	8 (1.7%)	8 (2.4%)			2 (4.7%)	0 (0.0%)		
**AJCC stage**								
I	102 (21.2%)	40 (12.0%)	11.59	0.00	11 (25.6%)	24 (12.3%)	4.94	0.03
II–IV	369 (52.9%)	286 (57.5%)			31 (53.5%)	166 (64.1%)		
N/A	11 (2.3%)	6 (1.8%)			1 (2.3%)	1 (0.5%)		
**Metastasis** status								
M0	390 (80.9%)	269 (81.0%)	0.54	0.46	39 (90.7%)	170 (87.2%)	14.67	0.00
M1	8 (1.7%)	8 (2.4%)			4 (9.3%)	4 (2.1%)		
N/A	84 (17.4%)	55 (16.6%)			0 (0.0%)	21 (10.8%)		
**PR**								
Negative	57 (11.8%)	66 (19.9%)	10.10	0.00	34 (79.1%)	186 (95.4%)	124	0.00
Positive	424 (88.0%)	264 (79.5%)			9 (20.9%)	7 (3.6%)		
N/A	1 (0.2%)	2 (0.6%)			0 (0.0%)	2 (1.0%)		
**HER2**								
Negative	381 (79.0%)	219 (66.0%)	20.21	0.00	32 (74.4%)	142 (72.8%)	0.01	0.92
Positive	60 (12.4%)	83 (25.0%)			8 (18.6%)	34 (17.4%)		
N/A	41 (8.5%)	30 (9.0%)			3 (7.0%)	19 (9.7%)		

*Note*. The data of 1052 BC samples with z‐scores of mRNA level were from the TCGA online database (www.cbioportal.org). *p* Value was calculated using the Pearson χ2 test. The percentages of each subgroup were described in brackets.

Abbreviations. AJCC, American Joint Committee on Cancer; HER2, Human Epidermal Growth Factor Receptor 2; PR, Progesterone Receptor; N/A, unavailable.

**FIGURE 5:**
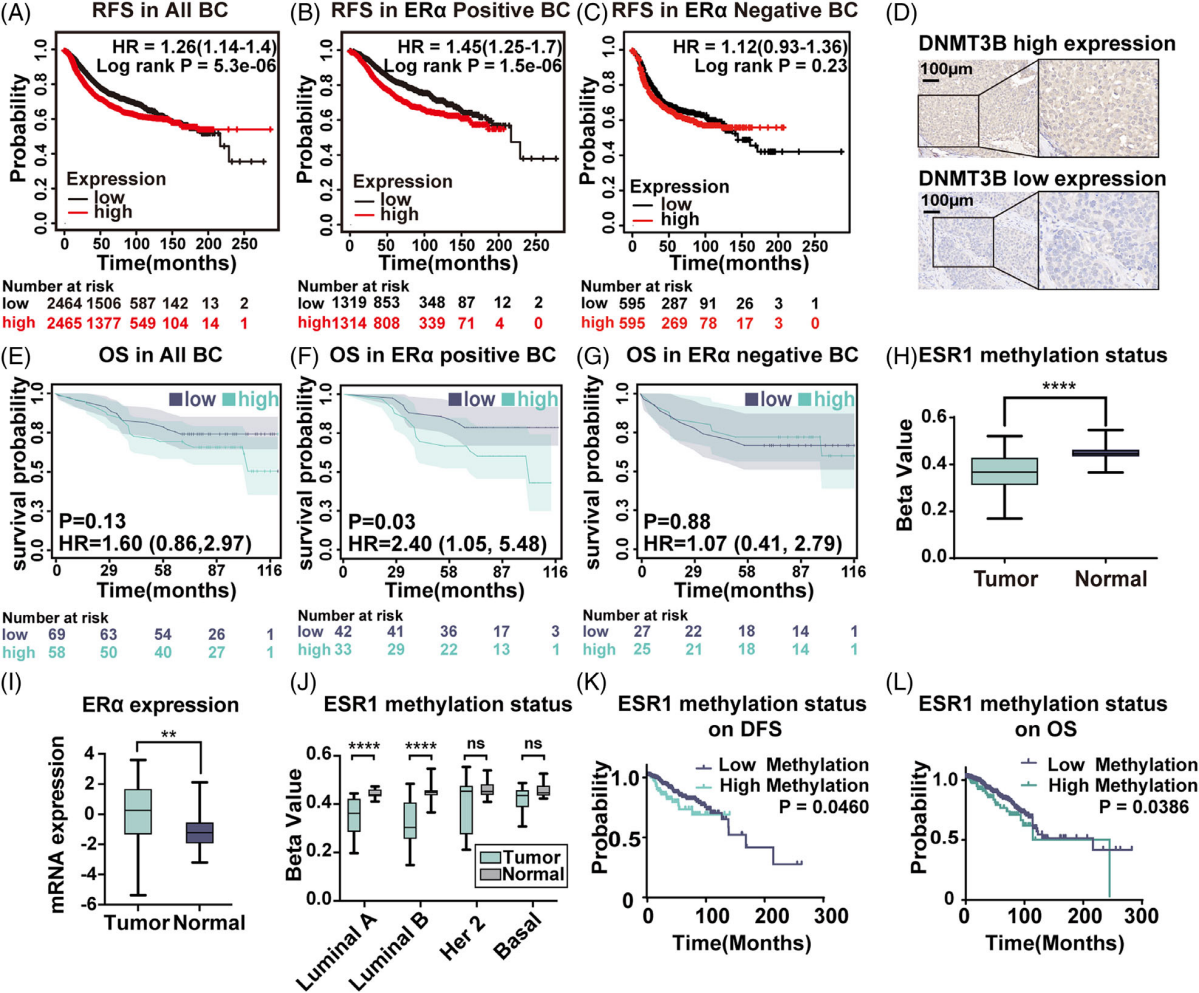
The prognostic value of DNMT3B expression and *ESR1* methylation status to BC patients. (A‐C) Kaplan–Meier survival analysis of RFS for all BC patients (n = 3951, A), ERα‐positive BC patients (n = 1402, B), ERα‐negative BC patients (n = 548, C) with high and low mRNA expression of DNMT3B, generated on the website: www.kmplot.com. (D) Representative microscopic images of DNMT3B high expression and DNMT3B low expression samples. Magnification, 400×. (E‐G) OS curve for all BC cancer patients (n = 127, E), ERα‐positive cancer patients (n = 75, F), ERα‐negative cancer patients (n = 52, G) with high and low expression of DNMT3B based on IHC score. (H) Comparison of *ESR1* methylation levels in 92 matched BC and adjacent normal tissue pairs. (I) Comparison of ERα‐expression levels in 92 compared BC and adjacent normal tissue pairs. (J) Comparison of *ESR1* methylation levels of matched pairs of BC and adjacent normal tissues in different BC subtypes (Luminal A: n = 29, Luminal B: n = 26; Her 2: n = 12; Basel: n = 12) (K) Kaplan–Meier survival analysis of DFS for BC patients (n = 788) with high and low levels of *ESR1* methylation (L) Kaplan–Meier survival analysis of OS for BC patients (n = 788) with high and low levels of *ESR1* methylation. RFS, relapse‐free survival; OS, overall survival; DFS, disease‐free survival.

To investigate the predictive value of DNMT3B expression in BC patients, we obtained prognostic‐related information from the website www.kmplot.com. Progression‐free survival curves were generated for all BC cases, ERα‐positive, and ERα‐negative patients. During 250 months of following‐up, we found elevated expression of DNMT3B mRNA in BC was associated with unfavorable relapse‐free survival (RFS) in all BC patients (hazard ratio [HR] = 1.26, log‐rank *p* = 5.3×10^−6^, Figure 5A), especially in ERα‐positive BC (HR = 1.45, *p* = 1.5×10^−6^, Figure 5B). There was no association between DNMT3B mRNA expression and RFS (Figure 5C) in ERα‐negative patients. Besides, we performed IHC staining of DNMT3B on a tissue microarray (TMA) of 140 human breast tumor specimens. And 13 cases with missing ERα expression data or unsuccessful staining were excluded from our analysis. Of the remaining 127 patients, 58 were DNMT3B high‐expressed, and 69 were DNMT3B low‐expressed, according to the grading criteria described in Section 4.14. Typical microscope images of DNMT3B are shown in Figure 5D. Overall survival (OS) curves were generated for all BC, ERα‐positive, and ERα‐negative patients. For ERα‐positive BC patients, high levels of DNMT3B indicated significantly worse outcomes (HR = 2.40, *p* = 0.03, Figure 5F). No predictive value of DNMT3B was found in ERα‐negative (HR = 1.07, *p* = 0.88, Figure 5G) and all BC patients (HR = 1.60, *p* = 0.13, Figure 5E). The predicting value of DNMT3B in ERα‐positive BC patients obtained from immunohistochemistry analysis was consistent with RFS results based on the RNA‐seq data from www.kmplot.com. In summary, these data indicated DNMT3B seemed to play a more significant role in ERα‐positive BC, which has not been reported.

### 2.6 ESR1 methylation was low in BC patients, and high ESR1 methylation predicted an unfavorable prognosis

To investigate the potential role of *ESR1* methylation in BC, we first analyzed its expression level in 92 pairs of BC and matched adjacent normal tissues from the TCGA database. The *ESR1* region has 63 CpG‐related probes, with 47 probes identifying CpG sites in the promoter. DNA methylation at the CpG site was measured as beta‐values. Our results showed that the mean beta‐value of *ESR1* methylation in BC patients was 0.37, while it was 0.45 in matched normal tissues (*p* < 0.0001, Figure 5H). On the contrary, the ERα mRNA expression level in tumor tissue was higher than in normal tissue (*p* = 0.001, Figure 5I). To further compare *ESR1* methylation status in different BC subtypes, we utilized data from 79 pairs of BC with complete clinical information. In Luminal A and Luminal B subtypes, the *ESR1* methylation level in tumor tissue was significantly lower than in the matched normal tissues (Luminal A: *p* < 0.0001, Luminal B: *p* < 0.0001, Figure 5J). However, no significant difference was found in HER2 and basal‐like subtypes. These results corresponded to the theory that *ESR1* methylation resulted in ERα suppression.

To evaluate the correlation between *ESR1* methylation status and clinicopathological features in BC, the selected 788 patients with complete methylation expression data were divided into a high methylation (670 cases) and a low methylation group (118 cases) by the best cutoff, as mentioned above. There was no apparent relationship between *ESR1* methylation and age or lymph node status. In comparison, the *ESR1* high methylation group had a significantly higher percentage of T4 tumors (5.1 to 2.7%; *p* < 0.001), distant metastasis status (4.2 to 1.2%; *p* < 0.001), American Joint Committee on Cancer (AJCC) stage IV (3.4 to 1.0%; *p* < 0.001), ERα negative (78.8 to 11.5%; *p* < 0.001), PR negative (83.1 to 21.0%; *p* < 0.001), HER2 positive (21.2 to 12.8%; *p* < 0.001), invasive ductal carcinoma (86.4 to 63.9%; *p* < 0.001) BC patients than the *ESR1* low methylation group (Table 2).

**TABLE 2:** Relationship between *ESR1* methylation and clinicopathological parameters in BC patients.

	ESR1 Methylation (%)			
Features	Low methylation (n = 670)	High methylation (n = 118)	χ^2^	*p*
**Age**				
≤50	215 (32.1%)	36 (30.5%)	0.13	0.72
>50	453 (67.6%)	82 (69.5%)		
No Data	2 (0.30%)	0 (0%)		
**Tumor size**				
T1–T3	648 (96.7%)	112 (94.9%)	14.25	0.00015968
T4	18 (2.7%)	6 (5.1%)		
No Data	4 (0.6%)	0 (0%)		
**Nodal status**				
N0–N2	611 (91.2%)	106 (89.8%)	0.042	0.84
N3	50 (7.5%)	8 (6.8%)		
No data	9 (1.3%)	4 (3.4%)		
**AJCC stage**				
I—III	654 (97.6%)	111 (94.1%)	13.99	0.000184247
IV	7 (1.0%)	4 (3.4%)		
No Data	9 (1.3%)	3 (2.5%)		
**Metastasis status**				
M0	524 (78.2%)	94 (79.7%)	24.21	0.000000862
M1	8 (1.2%)	5 (4.2%)		
No Data	138 (20.6%)	19 (16.1%)		
**ER**				
Negative	77 (11.5%)	93 (78.8%)	270.46	9.01E‐61
Positive	554 (82.7%)	19 (16.1%)		
No Data	39 (5.8%)	6 (5.1%)		
**PR**				
Negative	141 (21.0%)	98 (83.1%)	187.49	1.93E‐41
Positive	488 (72.8%)	13 (11.0%)		
No Data	41 (6.1%)	7 (5.9%)		
**HER2**				
Negative	484 (72.2%)	80 (67.8%)	4.91	0.03
Positive	86 (12.8%)	25 (21.2%)		
No Data	100 (14.9%)	13 (11.0%)		
**Cancer Type**				
IDC	428 (63.9%)	102 (86.4%)	25.17	0.000000524
ILC	177 (26.4%)	7 (5.9%)		
Others	65 (9.7%)	9 (7.6%)		

*Note*. The data for 788 BC samples with a beta value of *ESR1* methylation were from the TCGA database. *p* Value was calculated using the Pearson χ*^2^* test.

Abbreviations. AJCC, American Joint Committee on Cancer; ER, Estrogen Receptor; HER2, Human Epidermal growth factor Receptor 2; IDC, Invasive Ductal Carcinoma; ILC, Invasive Lobular Carcinoma; PR: Progesterone Receptor.

We next examined the prognostic effect of *ESR1* methylation for BC patients. A total of 788 samples with complete methylation expression data from 1108 BC samples obtained from TCGA were analyzed. A beta value of 0.7472 obtained by the X tile was used to distinguish the low and high methylation groups. High *ESR1* methylation was associated with a worse prognosis of disease‐free survival (DFS) (HR = 1.844, 95% confidence interval [CI]: 1.011 to 3.363, *p* = 0.0460) and OS (HR = 1.746, 95% CI: 1.030 to 2.963, *p* = 0.0386) in all BC patients (Figure 5K and L).

## 3 DISCUSSION

ERα is both a prognostic and predictive marker in BC, and hypermethylation of its promoter is a crucial gene‐silencing mechanism in ERα‐negative BC.^32^ In ERα‐positive BC cell lines, such as MCF‐7, T47D, and ZR75‐1, the *ESR1* promoter is considered nonmethylated.^8^, ^33^ Previously, a few EMT‐related transcriptional factors, such as Twist and ZEB1, were reported to induce hypermethylation of the *ESR1* promoter and result in transcriptional repression of ERα. Our group and others have shown that SNAI2 could transcriptionally repress *ESR1* both at ERα‐positive and ‐negative BC cells.^34^, ^35^ However, how SNAI2 regulates *ESR1* methylation is still unclear.

In the present study, we found that SNAI2 positively correlated with *ESR1* methylation, especially in ERα‐positive BC patients. We also showed that overexpression of SNAI2 promoted *ESR1* methylation in MCF‐7 cells, while knockdown of SNAI2 barely affected *ESR1* methylation in MDA‐MB‐231 cells. First, we used antisense oligonucleotides to identify which DNMTs may participate in *ESR1* methylation in SNAI2‐overexpressed MCF‐7 cells. Then, we applied Co‐IP, ChIP‐qPCR, and dual‐luciferase reporter assay to further confirm the binding of SNAI2 with DNMT3B, illustrating the impact of the SNAI2‐DNMT3B complex on *ESR1* transcriptional activity. Thus, in this study, we proposed a new molecular mechanism that SNAI2 promotes *ESR1* methylation to repress *ESR1* transcription by recruiting DNMT3B in ERα‐positive BC.

Concerning the function of *ESR1* methylation, it had been well documented that the *ESR1* methylation predicted endocrine resistance in previous research.^17^, ^36^, ^37^ However, are there any other effects of *ESR1* methylation in both cells and humans? By searching and analyzing GO, KEGG, and GSEA databases, we identified enriched cellular components and pathways tightly associated with cell junctions and adhesions, suggesting that *ESR1* methylation might promote cancer metastasis. Among the many mechanisms of BC metastasis reported, including immunosuppressive microenvironments and autophagy,^38^, ^39^ EMT is the most straightforward process to stimulate metastasis at the cellular level. Our group^40^ and other research teams^25^, ^29^, ^41^ ^,^ ^42^ demonstrated that ERα could suppress EMT, cell migration, invasion, and metastasis in BC patients. Taken together, those findings prompted us to investigate the role of *ESR1* methylation on EMT. In the functional studies conducted using the MCF‐7 cell line, we found that SNAI2 overexpression caused methylation of *ESR1* promoter and promoted EMT. Moreover, when we overexpressed SNAI2 and knocked down DNMT3B together, not surprisingly, DNMT3B silencing partially rescued ectopic SNAI2 expression‐induced EMT, indicating that EMT induced by SNAI2/DNMT3B was indeed mediated by *ESR1* methylation.

Very little is known about the effect of DNMT3B in solid neoplasms including BC. Leveraging the large‐scale clinical data from TCGA, we found that DNMT3B overexpression was strongly associated with large tumor size, lymph node involvement, and advanced AJCC stage, especially in ERα‐positive BC patients.

In addition to the relationship of DNMT3B with clinicpathological factors, we also determined its prognostic significance in ERα‐positive BC patients. We found that increased expression of DNMT3B mRNA was significantly correlated with poor RFS in ERα‐positive BC patients, while no prognostic value was discovered in ERα‐negative BC patients. In the immunohistochemical (IHC) test of TMA, we found high DNMT3B expression predicted worse OS in only ERα‐positive BC patients. Our results were consistent with the finding of Igor Girault's group that DNMT3B overexpression was associated with shorter RFS in a subgroup of patients who had received adjuvant hormone therapy.^43^ In contrast, Yu's study demonstrated that DNMT3B was not associated with DFS or OS in BC patients.^44^ This discrepancy could be due to different sources of breast tissue samples. In addition, testing methods to evaluate DNMT3B expression level (immunohistochemistry or real‐time RT‐PCR assays) and classification mode (DNMT3B negative/positive or DNMT3B low/high) also impacted the results.

The reports of the levels of *ESR1* methylation in BC and normal breast tissues were different. Raad S. Gitan found an increased DNA hypermethylation of the *ESR1* CpG island in breast tumors compared to normal controls by methylation‐specific oligonucleotide microarray.^33^ In contrast, the research from Hoque MO showed similar levels and frequency of ESR1 methylation in both normal and neoplastic breast tissues by MSP.^45^ Except for breast tissues, methylated *ESR1* levels in serum or plasma were also investigated and found to be higher in BC than in healthy control and benign breast disease.^46^, ^47^ Our study and Chao Hu's research^48^ found that invasive breast carcinoma showed more hypomethylation of *ESR1* than healthy tissues. The above inconsistency may be attributed to the different specimen sources (breast tissues or serum/plasma), different test platforms (MSP or methylation array), or selective bias caused by limited clinical specimens.

The correlation between *ESR1* methylation and clinicopathological characteristics has not been discussed yet. With the help of the large‐scale clinical data and expression level information from TCGA, we found that the high *ESR1* methylation group had a strong correlation with bigger tumor size, distant metastasized status, advanced clinical stage, negative ERα and/or PR, positive HER2 status, and higher invasive ductal carcinoma percentage in BC patients. It seemed that *ESR1* methylation promoted tumor progression. Our research indeed showed that higher *ESR1* hypermethylation was significantly associated with shorter OS and RFS.

However, there are still some limitations to our study. The underlying mechanisms of how SNAI2 recruited and bound DNMT3B were still ambiguous. We did not verify whether SNAI2 interacted with DNMT3B directly or indirectly. The specific binding sites or intermediate factors are still unclear and need further exploration.

**FIGURE 6:**
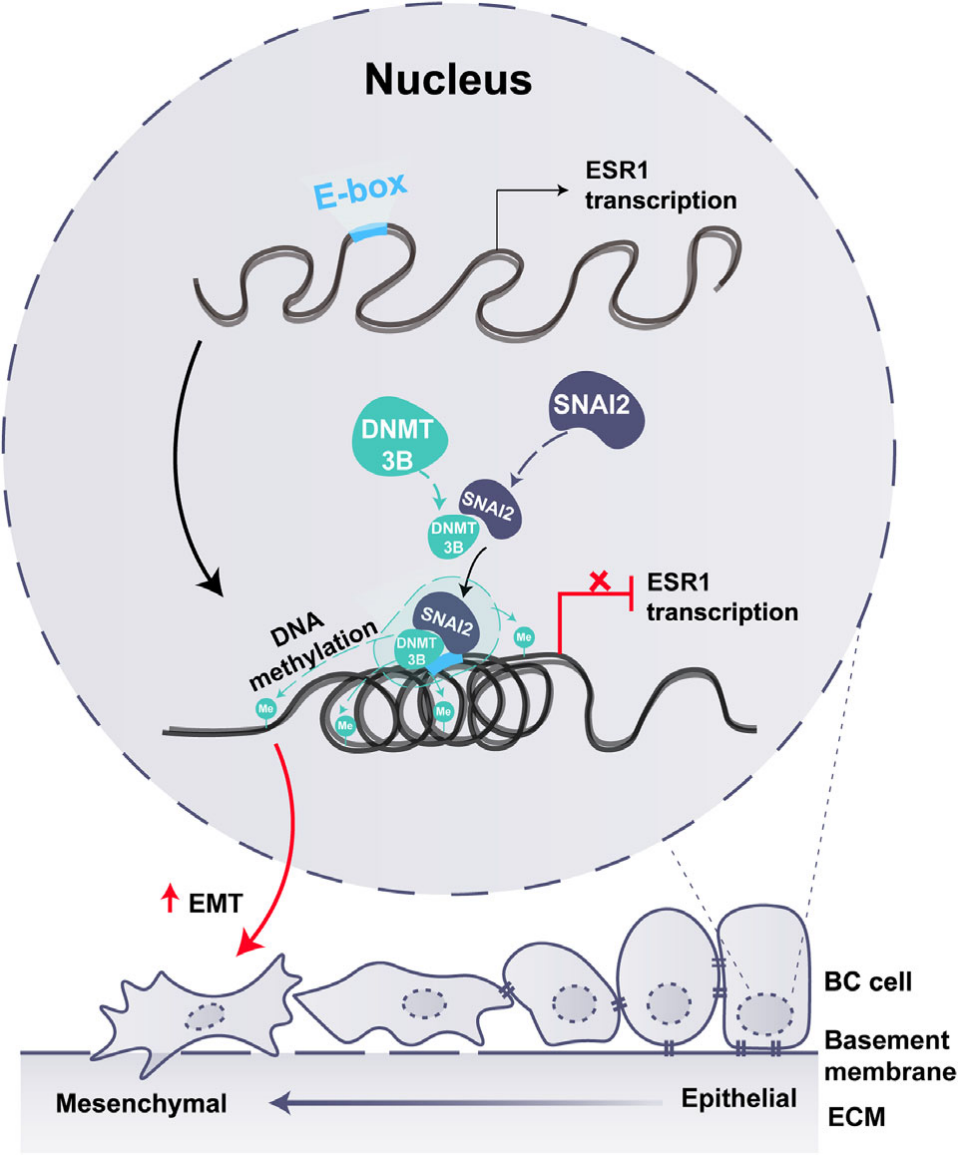
A schematic view of DNMT3B recruitment by SNAI2 for repressing ERα expression and promoting EMT. In ERα‐positive BC cell, SNAI2 recruited DNMT3B to form a SNAI2‐DNMT3B combination and bound on the E‐box region of the *ESR1* promoter to stimulate promoter methylation. The elevated *ESR1* methylation resulted in a condensed chromatin state, transcriptional repression, and gene silencing of *ESR1*. Besides, SNAI2‐DNMT3B‐*ESR1* also significantly facilitated the process of EMT and promoted the invasiveness of ERα‐positive BC cells. BC cells, breast cancer cells; EMT, Epithelial–Mesenchymal Transition; ECM, extra cellular matrix.

Taken together, our study demonstrated that *ESR1* promoter methylation was most likely induced by the SNAI2‐DNMT3B complex and resulted in enhanced EMT in ERα‐positive BC (Figure 6). Moreover, high *ESR1* methylation was associated with tumor progression and predicted a poor prognosis. Our study identified critical molecular mechanisms for epigenetic regulation of *ESR1* in ERα‐positive BC cells and provided a potential target for BC treatment to prevent tumor metastasis.

## 4 MATERIALS AND METHODS

### 4.1 Online data acquisition and analysis

Gene expression, Illumina 450 k Human Methylation platform array data, and clinical information of BC patients and cancer‐adjacent normal tissues were sourced and downloaded from the TCGA online database (www.cbioportal.org). Methylation values are quantitatively represented as beta‐values, calculated by taking the mean percentage methylation of all probes in the promoter region, ranging from 0 (unmethylated) to 1 (completely methylated).^49^

Online database^50^ (http://kmplot.com/analysis/index.php?p = service&cancer = breast) was utilized to assess the association between DNMT3B expression and RFS.

### 4.2 Gene ontology (GO) enrichment, Kyoto encyclopedia of genes and genomes (KEGG) pathway enrichment, and gene set enrichment analysis (GSEA)

GO enrichment and KEGG pathway enrichment analysis of *ESR1* methylation‐related mRNAs were conducted using the cluster profile R package (R 3.6.3). Only the top ten most significant enriched terms were listed. GSEA analysis was performed by generating a preranked list of genes and inputting the index into the GSEA desktop application (http://software.broadinstitute.org/gsea/downloads.jsp).

### 4.3 Cell culture

BC cell lines, including MCF‐7, T47D, MDA‐MB‐231, and human embryonic kidney HEK293T cells, were purchased from the Chinese Type Culture Collection (Chinese Academy of Sciences). Cells were cultured in DMEM medium supplemented with 10% fetal bovine serum (FBS), 100 units/mL penicillin, and 100 μg/mL streptomycin. The FBS and penicillin/streptomycin were purchased from Thermo Fisher Scientific . The cells were incubated at 37°C in a 5% CO_2_ humidified atmosphere.

### 4.4 Plasmids, siRNA, and transfection

pcDNA3.1‐SNAI2‐Flag, pcDNA3.1 and pcDNA3.1‐ΔN‐SNAI2 lacked the SNAG domain, *ESR1* promoter 2‐luc, specific siRNAs targeting SNAI2, MCF‐7 stable cells (MCF‐7SNAI2 or MCF‐7NC) and MDA‐MB‐231 stable cells (MDA‐MB‐231shSNAI2 or MDA‐MB‐231shNC) were described in our previous study.^34^ pcDNA3/Myc‐DNMT3B was a gift from Arthur Riggs (Addgene plasmid # 35522).^51^ DNMT3B and the scrambled siRNA were purchased from Santa Cruz Biotechnology. The transfection was performed using Lipofectamine 2000 (Thermo Fisher Scientific) when cell confluent reached 70−90%, following the manufacturer's instructions.

### 4.5 Western blotting, reverse transcription, and RT‐PCR

Methods for protein extraction, Western blotting, RNA purification, reverse transcription, and real‐time PCR analysis have been previously outlined.^52^ The detailed information regarding the antibodies and primers utilized in this study are given in Supporting Information Tables S1 and S2.

### 4.6 Methylation‐specific PCR

According to the vendor's instructions, genomic DNA was extracted and bisulfite modified using the EpiTect Bisulfite Kit (Qiagen). After PCR, the product was photographed by 2% agarose gel electrophoresis. The primers used in MSP are also provided in Supporting Information Table S2.

### 4.7 Bisulfite sequencing PCR

The procedures of the DNA extraction and bisulfite modification followed similar protocols to MSP. The PCR amplicon was then cloned into the pMD18‐T vector (TaKaRa) and transformed into *Escherichia coli* strain DH5α for sequencing. The methylation levels were calculated as the ratio of methylated cytosine to the total number of cytosine. The primers used for BSP are listed in Supporting Information Table S2.

### 4.8 Co‐immunoprecipitation assay

HEK293T cells or MCF‐7 cells were seeded in a 100‐mm dish and co‐transfected with corresponding plasmids (15 μg each). Cells were then lysed using Western blot and IP cell lysis buffer (Beyotime), supplemented with phenylmethanesulfonyl fluoride (Beyotime) protease inhibitor. Cell lysates were incubated with anti‐Flag, anti‐Myc, or anti‐IgG antibodies overnight at 4°C, followed by a 4‐h incubation with protein A + G Plus Agarose at 4°C. Samples were then washed and resolved by 2× electrophoresis sample buffer with 2‐mercaptoethanol. The effectiveness of co‐IP was verified by Western blot.

### 4.9 Chromatin immunoprecipitation assay

Approximately 2 × 10^7^ MCF‐7NC or MCF‐7SNAI2 cells were lysed and sonicated, then incubated with anti‐SNAI2 or anti‐DNMT3B antibodies, along with Pierce™ Protein A/G magnetic beads (Thermo Fisher Scientific). After washing and reverse‐cross‐linking, the purified DNA samples were assessed using real‐time qPCR. The primers used in ChIP‐qPCR are supplied in Supporting Information.

### 4.10 Luciferase reporter assay

MCF‐7 cells were co‐transfected with *ESR1* promoter 1‐Luc plasmid (−2410 to −1410 bp, covering first three putative binding sites) or *ESR1* promoter 2‐Luc plasmid (−910 to +90 bp, covering the fourth through seventh binding sites), in addition to pRL‐SV40 and SNAI2 or mutant ΔN‐SNAI2 (without SNAG domain) with or without DNMT3B knocking down. Transfection efficiency was normalized using Renilla luciferase activity.

### 4.11 CCK8 assay and colony formation assay

For the CCK8 assay, we followed the manufacturer's (Beyotime) instructions. MCF‐7 cells were seeded in a 96‐well microplate at a density of 5 × 10^3^ cells per well and cultured for varying durations (1 d, 2 d, 3 d, and 4 d) before the CCK8 assay.

For the colony formation assay, 5 × 10^2^ MCF‐7NC or MCF‐7SNAI2 cells with or without DNMT3B knockdown were cultured in a six‐well plate per well for 2 weeks. After fixing and staining, colonies were counted via a microscope.

### 4.12 Wound healing assay, cell migration, and invasion assays

Wound healing assays, cell migration, and invasion assays were performed according to both the manufacturer's instructions and previous work.^53^

### 4.13 Tissue samples

TMA containing samples from 140 human BC patients was purchased from Shanghai Outdo Biotech Company (CAT No. HbreD140Su05), which included data on expression levels of ER, PR, HER2, and survival data of each sample.

### 4.14 Immunohistochemical analysis

According to the manufacturer's protocol, IHC analysis was performed using an UltraSensitiveTM SP IHC kit (Maxim). Sections were boiled in retrieval solutions to expose antigens after deparaffinization and rehydration and then incubated with an anti‐DNMT3B antibody (see Supporting Information Table S1). Subsequently, slides were counterstained with hematoxylin, dehydrated, mounted, and examined under a microscope. Two expert pathologists assessed the staining area and intensity, generating an IHC score based on these parameters. The size of stained tumor cells was scored as follows: 0 (0–5%), 1 (6–25%), 2 (26–50%), 3 (51–75%), and 4 (>75%). Staining intensity was scored as follows: 0 (no staining), 1 (light yellow), 2 (yellow), and 3 (brown). The final IHC score was obtained by multiplying these two subscores and was classified into high (>6) and low (⩽6) expressions.

### 4.15 Statistical analysis

SPSS software (V22) and GraphPad Prism 9.0 were used for statistical analysis. The differences between the two groups were analyzed using the Student's *t*‐test, while those among three or more groups were calculated by one‐way ANOVA. The Pearson correlation coefficient, Pearson's chi‐square test, or Fisher's exact test was used to evaluate the correlation between *ESR1* methylation and SNAI2 expression or BC patients’ clinicopathological parameters. Survival analysis was conducted through the Kaplan–Meier method, with statistical significance determined by the Log‐rank test. A *p* value below 0.05 was deemed statistically significant.

## AUTHOR CONTRIBUTIONS

Ji‐Wei Li and Qiu‐Min Deng performed experiments, analyzed data, and wrote the manuscript. Jian‐Ling Zhu and Xiao‐Long Wei contributed to IHC staining and analysis. Min Wei was responsible for generating stable cell lines. Xiao‐Yi Hu contributed to the BSP analysis. Lin‐Ling Lin conducted the online data analysis. Hong‐Yu Chen and Zhong Luo analyzed and visualized the data. Yong‐Qu Zhang performed the MSP analysis. Kang‐Liang Lou and Yi‐Yang Gao performed EMT‐related functional experiments. Guo‐Jun Zhang and Jing‐Wen Bai designed the project, supervised all experiments, and analyzed the results. All authors read and approved the final manuscript.

## CONFLICT OF INTEREST STATEMENT

The authors declare no conflict of interest.

### ETHICS APPROVAL

The use of human breast cancer tissue samples was approved by the Ethics Committee of Shanghai Outdo Biotech Company (SHYJS‐CP‐1901002).

## Supporting information

Supporting information

## Data Availability

The data that support the findings of this study are available from the corresponding author upon reasonable request.
