# Supplementary material for: Methylation of ESR1 promoter induced by SNAI2–DNMT3B complex promotes epithelial–mesenchymal transition and correlates with poor prognosis in ERα‐positive breast cancers
Source: MedComm (2020). 2023 Oct 24;4(6):e403. doi: 10.1002/mco2.403 (PMC10594044; doi:10.1002/mco2.403)
Supplement: Supplementary file 1 — Supporting information [file MCO2-4-e403-s001.docx]

**Methylation of ESR1 promoter induced by SNAI2–DNMT3B complex promotes epithelial-mesenchymal transition and correlates with poor prognosis in ERα positive breast cancers**

**Running title:** SNAI2 promotes ESR1 methylation and EMT in BC.

Ji-Wei Li^#1,7^, Qiu-Min Deng^#1,2,3,4^, Jian-Ling Zhu^8^, Wei Min^1,2,3,4^, Xiao-Yi Hu^7^, Hong-Yu Chen^1,2,3,4^, Zhong Luo^1,2,3,4^, Lin-Ling Lin^1,2,3,4^, Xiao-Long Wei^8^, Yong-Qu Zhang^1,2,3,4^, Kang-Liang Lou^1,2,3,4^, Yi-Yang Gao^1,2,3,4^, Guo-Jun Zhang^1,2,3,4,6^, Jing-Wen Bai*^1,2,3,5^

**Authors' Affiliations:**

^1^ Fujian Key Laboratory of Precision Diagnosis and Treatment in Breast Cancer, Xiang'an Hospital of Xiamen University, School of Medicine, Xiamen University, Xiamen, China, 361100

^2^ Xiamen Key Laboratory of Endocrine-Related Cancer Precision Medicine, Xiang'an Hospital of Xiamen University, School of Medicine, Xiamen University, Xiamen, China, 361100

^3^ Xiamen Research Center of Clinical Medicine in Breast and Thyroid Cancers, Xiang'an Hospital of Xiamen University, School of Medicine, Xiamen University, Xiamen, China, 361100

^4^ Department of Breast-Thyroid-Surgery and Cancer Center, Xiang'an Hospital of Xiamen University, School of Medicine, Xiamen University, Xiamen, China, 361100

^5^ Department of Oncology, Xiang'an Hospital of Xiamen University, School of Medicine, Xiamen University, Xiamen, China, 361100

^6^ Cancer Research Center of Xiamen University, School of Medicine, Xiamen University, Xiamen, China, 361100

^7^ Department of Respiratory, Critical Care and Sleep Medicine, Xiang’an Hospital of Xiamen University, School of Medicine, Xiamen University, Xiamen, 361100, P.R. China

^8^ Department of Pathology, the Cancer Hospital of Shantou University Medical College, China, 515031

^#^These authors contributed equally to this work. Ji-Wei Li: jiweili2019@stu.xmu.edu.cn, Qiu-Min Deng: dqmluck@163.com

^*^Correspondence to: Jing-wen Bai, MD, Ph.D., [baijingwen666@126.com](mailto:baijingwen666@126.com)

**
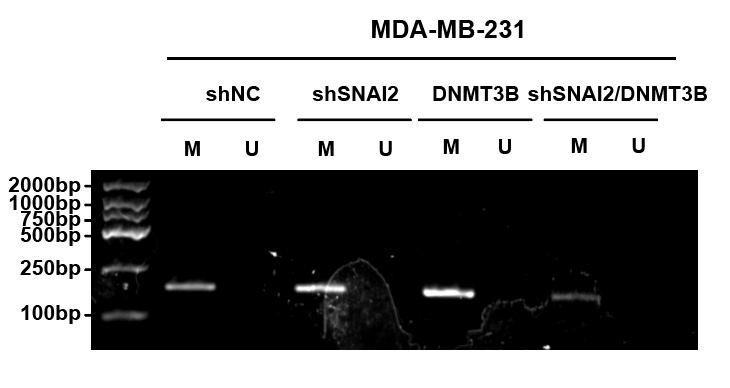
**

**Figure S1. MSP analysis for MDA-MB-231 shNC, MDA-MB-231 shSNAI2, MDA-MB-231 DNMT3B and MDA-MB-231 shSNAI2/DNMT3B cells.**

MSP analysis of methylation status of ESR promoter in MDA-MB-231 shSNAI2, MDA-MB-231 DNMT3B, MDA-MB-231 shSNAI2/DNMT3B and the control group. Transfections were performed in 6-cm dishes and the amount of the plasmid for overexpressing DNMT3B were 6ug per well.

**
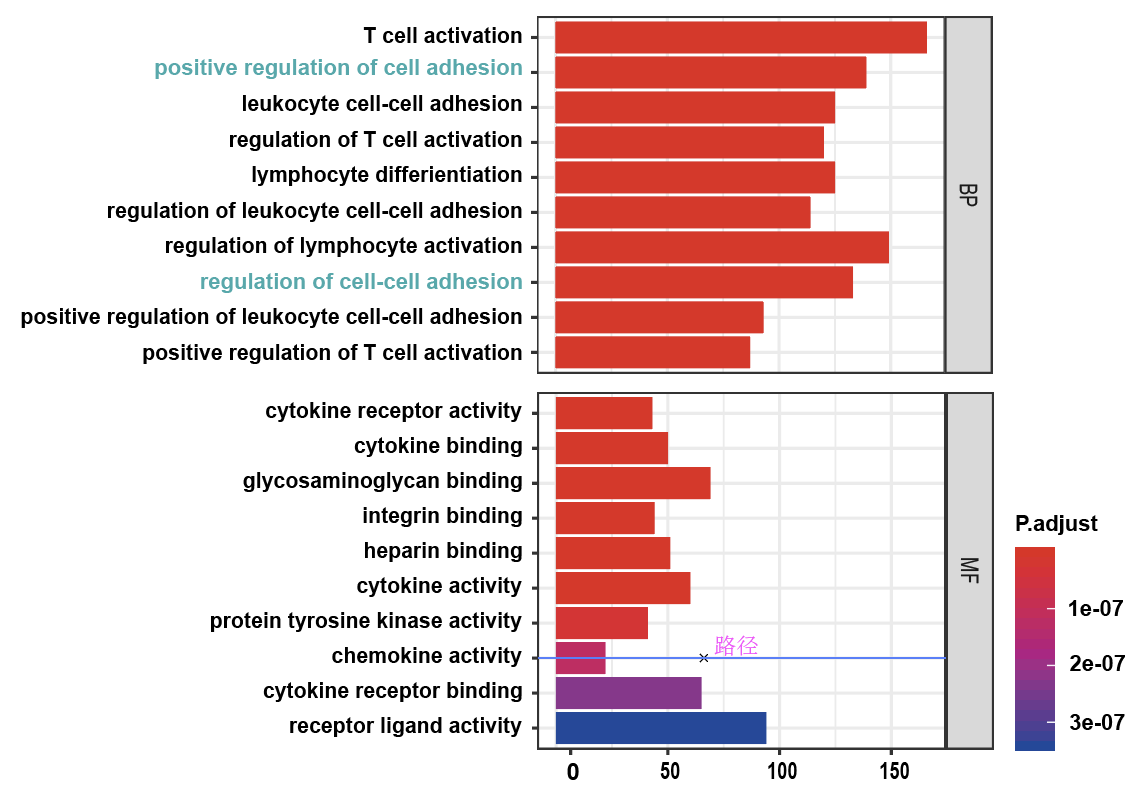
 Figure S2. GO analysis for ESR1 methylation-related genes.**

GO analysis of the biological process (upper) and molecular function (lower) analysis for ESR1 methylation-related genes whose mRNAs retain a |Pearson correlation coefficient| ≥ 0.3

**
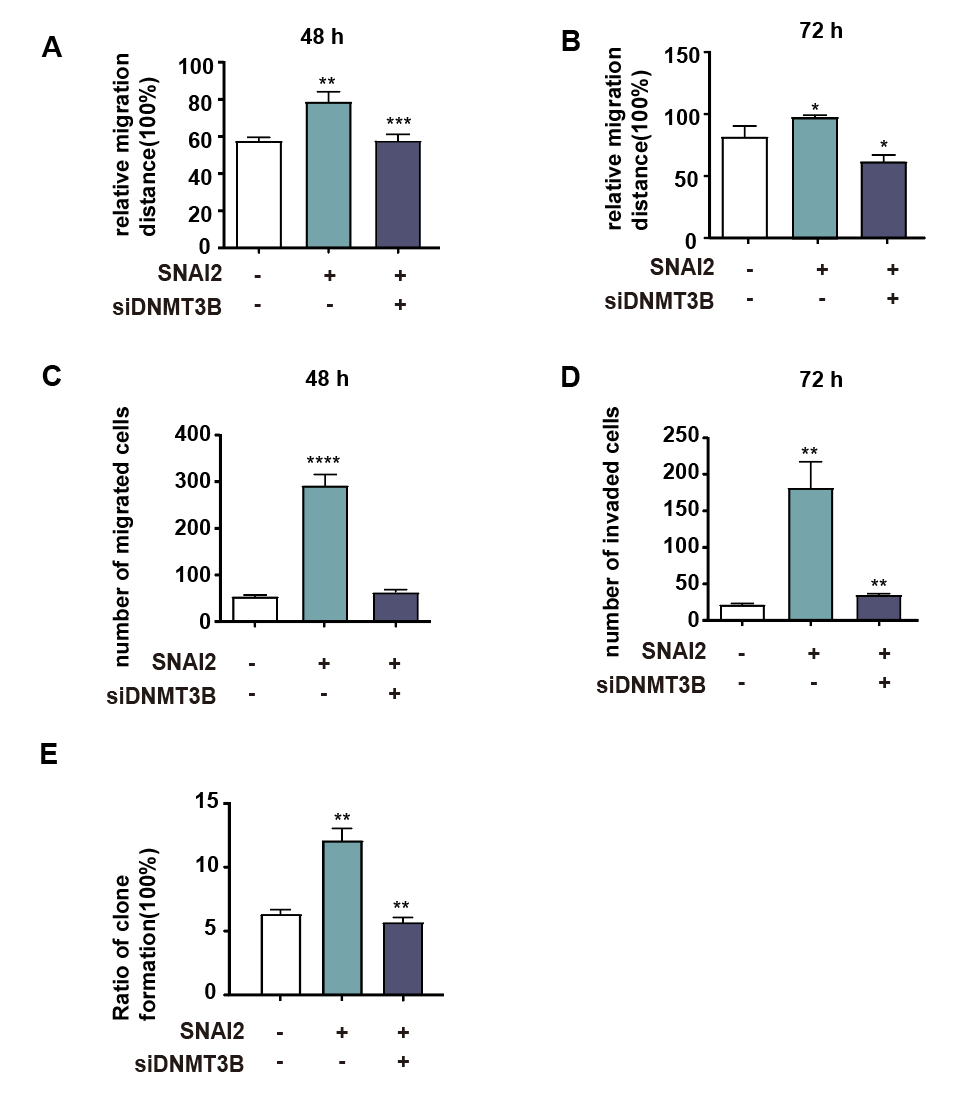
**

**Figure S3. Comparisons of relative migration distances, number of migrated cells, and ratios of clone formation after transfection.**

(A&B) Comparisons of relative migration distances of MCF7 cells, MCF-7 cells with SNAI2-overexpression, and SNAI2-overexpressed MCF7 cells with DNMT3B-knockdown after 48h (A) and 72h (B). (C&D) Comparisons of the number of migrated cells of MCF7 cells, MCF-7 cells with SNAI2-overexpression, and SNAI2-overexpressed MCF7 cells with DNMT3B-knockdown after 48h (C) and 72h (D). (E) Comparisons of ratios of clone formation of MCF7 cells, MCF-7 cells with SNAI2-overexpression, and SNAI2-overexpressed MCF7 cells with DNMT3B-knockdown

**Table S1. Antibodies used in this study**

| Antibody | Cat.No. | Company | Usage |
| --- | --- | --- | --- |
| SNAI2 | 9585S | Cell Signaling Technologies | WB; IP; ChIP |
| ERα  ER$\beta$ | 8644S  14007-1-AP | Cell Signaling Technologies  Proteintech | WB;  WB |
| DNMT3B  DNMT3B  DNMT1 | 57868S  26971-1-AP  5032 | Cell Signaling Technologies  Proteintech  Cell Signaling Technologies | WB; IP; ChIP  IHC  WB; IP |
| GAPDH | 51332 | Cell Signaling Technologies | WB |
| Flag | F3165 | Sigma-Aldrich | WB |
| β-actin | sc-47778 | Santa Cruz Biotechnology | WB |
| Goat anti-mouse IgG-HRP | sc-2005 | Santa Cruz Biotechnology | WB |
| Goat anti-rabbit IgG-HRP | sc-2301 | Santa Cruz Biotechnology | WB; IHC |

**Table S2. The sequence of primers used in ChIP-qPCR, RT-PCR, MSP, and BSP.**

| **Primers** | **Sequence** |
| --- | --- |
| **ChIP primers for ESR1 promoters** | |
| Binding sites 1 Fwd | GTCTGGTTTCCTGGTGCAAT |
| Binding sites 1 Rev | CAAATGCCTTACTGGCCCTA |
| Binding sites 2 Fwd | CAGCAAGTCTCCCCTCACTC |
| Binding sites 2 Rev | ATTCGGGAAGCAGCCAGTAG |
| Binding sites 3 Fwd | TGAAGTGCTTTTTGCATGTG |
| Binding sites 3 Rev | CCTTTATGGCCAGCAATCAT |
| Binding sites 4 Fwd | TGGACCAGACCGACAATGTA |
| Binding sites 4 Rev | GTTCATGCCTTCCACAGGTT |
| Binding sites 5 Fwd | AGTTCCCCCAGCTGCTAAAT |
| Binding sites 5 Rev | ACCCTGGGGAGGACTACACT |
| Binding sites 6 Fwd | CAGCAGCGACGACAAGTAAA |
| Binding sites 6 Rev | TGCATTACAAAGGTGCTGGA |
| Binding sites 7 Fwd | ACAGTGTAGTCCTCCCCAGG |
| Binding sites 7 Rev | TTGTCGTCGCTGCTGGATAG |
| **PCR primers** | |
| ERα Fwd | TGCTTCAGGCTACCATTATGGA |
| ERα Rev | TGGCTGGACACATATAGTCGTT |
| SNAI2 Fwd | AAGCATTTCAACGCCTCCAAA |
| SNAI2 Rev | GGATCTCTGGTTGTGGTATGACA |
| DNMT3B Fwd | GTGCAGTACCTCAGCCCAAAG |
| DNMT3B Rev | CCGAGCCCAGGGATCAG |
| β-actin Rev | AGCGAGCATCCCCCAAAGTT |
| β-actin Fwd | GGGCACGAAGGCTCATCATT |
| E-cadherin Fwd | AAAGGCCCATTTCCTAAAAACCT |
| E-cadherin Rev | TGCGTTCTCTATCCAGAGGCT |
| N-cadherin Fwd | ACAGTGGCCACCTACAAAGG |
| N-cadherin Rev | CCGAGATGGGGTTGATAATG |
| Vimentin Fwd | GAGAACTTTGCCGTTGAAGC |
| Vimentin Rev | GCTTCCTGTAGGTGGCAATC |
| **BSP primers** | |
| ESR1 BSP Fwd | AAGTAAAGTAAAGTTTAGGGAAGTTGTT |
| ESR1 BSP Rwd | CTAATACAATAAAACCATCCCAAATAC |
| **MSP primers** | |
| ESR1 MSP M Fwd | CGAGTTGGAGTTTTTGAATC |
| ESR1 MSP M Rwd | ACCGATCTAACCGTAAACCT |
| ESR1 MSP U Fwd | AATGAGTTGGAGTTTTTGAATT |
| ESR1 MSP U Rwd | AAACCAATCTAACCATAAACCT |
| Note: Fwd, forward; Rev, reverse | |
